# Microstructural Alterations of the Corpus Callosum in Patients with First-Episode Schizophrenia Revealed by NODDI: Dissociation Between Neurite Density and Orientation Dispersion in the Splenium

**DOI:** 10.3390/bioengineering13050527

**Published:** 2026-04-30

**Authors:** Qiuping Ding, Qiqi Tong, Hongjian He, Bin Gao, Ling Xia

**Affiliations:** 1 Center for Brain Imaging Science and Technology, Key Laboratory for Biomedical Engineering of Ministry of Education, College of Biomedical Engineering and Instrument Science, Zhejiang University, Hangzhou 310058, China; 2 Research Center for Data Hub and Security, Zhejiang Lab, Hangzhou 310008, China; 3 School of Physics, Zhejiang University, Hangzhou 310058, China; 4 Department of Psychiatry, The Second Affiliated Hospital, Zhejiang University School of Medicine, Hangzhou 310030, China

**Keywords:** diffusion-weighted imaging, schizophrenia, corpus callosum, splenium, NODDI, neurite density index, orientation dispersion index, PANSS, negative symptoms

## Abstract

Background: Microstructural abnormalities of the corpus callosum (CC) are a consistent finding in schizophrenia, yet conventional diffusion tensor imaging (DTI) metrics provide limited biological specificity. Neurite orientation dispersion and density imaging (NODDI) can disentangle the neurite density index (NDI) and the orientation dispersion index (ODI), providing indirect, model-based markers of white matter microstructure in vivo. Methods: We applied NODDI to diffusion-weighted MRI data in patients with first-episode schizophrenia (FES) and matched healthy controls (HCs). The CC was used as a mask and subdivided into the genu (GCC), body (BCC), and splenium (SCC). Group differences in z-scores of the NDI and ODI were assessed using voxel-wise statistics within the CC and region of interest (ROI) analyses in the GCC, BCC, and SCC, controlling for age and sex. Associations between NODDI metrics and clinical symptoms were examined using the Positive and Negative Syndrome Scale (PANSS). Results: FES patients showed a significantly increased ODI in portions of the GCC, BCC, and SCC, as well as region-specific NDI alterations, with decreased NDI in parts of the SCC and increased NDI in sub-regions of the GCC/BCC (voxel-wise *p* < 0.05, FWE-corrected). ROI analyses confirmed a significant reduction in NDI *z*-scores in the SCC in FES patients compared with HCs (*p* = 0.009), whereas the ODI *z*-scores in the SCC did not differ significantly between groups (*p* = 0.124). Despite the absence of group-level ODI differences in the SCC, the SCC ODI was positively correlated with PANSS negative symptom scores in FES patients (*r* = 0.554, *p* = 0.002) and was also positively correlated with PANSS total scores in FES (*r* = 0.457, *p* = 0.014). This association remained significant in the region of the SCC after regressing out NDI from ODI (residual *z*_ODI), which was correlated with PANSS negative scores (*r* = 0.503, *p* = 0.006) and PANSS total scores (*r* = 0.474, *p* = 0.011), and the ODI/NDI ratio in the SCC was also correlated with negative symptom severity (*r* = 0.457, *p* = 0.014). Conclusions: Our findings suggest that, in the SCC, negative symptoms in schizophrenia are linked to altered neurite orientation dispersion under conditions of reduced neurite density. The dissociation between group-level NDI and ODI effects and their distinct relationship with psychopathology highlights the value of composite microstructural indices (e.g., residual *z*_ODI, ODI/NDI) for capturing clinically relevant white matter abnormalities.

## 1. Introduction

White matter abnormalities, particularly within the corpus callosum (CC), are among the most robust neuroimaging findings in schizophrenia [1,2]. Numerous diffusion tensor imaging (DTI) studies have reported reduced fractional anisotropy (FA) and increased mean diffusivity (MD) in the CC of schizophrenia patients, suggesting disrupted interhemispheric connectivity and impaired integration of information across cerebral hemispheres [1,3,4]. However, DTI metrics are inherently non-specific, as they conflate multiple microstructural features such as axonal density, myelination, and fiber orientation dispersion [1,5,6].

Neurite orientation dispersion and density imaging (NODDI) is an advanced diffusion MRI model that overcomes some limitations of DTI by explicitly modeling intracellular, extracellular, and isotropic diffusion compartments [6,7]. NODDI provides two key parameters: the neurite density index (NDI), reflecting the volume fraction of axons and dendrites, and the orientation dispersion index (ODI), reflecting the angular variability of neurites. In contrast to conventional DTI, which primarily captures the directionality and anisotropy of water diffusion and thus conflates several microstructural features, the NDI is designed to more directly reflect the structural density of neurites (axons and dendrites), providing a biologically more specific measure of tissue microstructure. These measures offer increased biological interpretability and have been successfully applied to characterize microstructural changes in schizophrenia and related psychotic disorders [8,9].

The CC is topographically organized into the genu (GCC), body (BCC), and splenium (SCC), each connecting distinct cortical regions and functions. Previous studies suggest that posterior callosal fibers, including the SCC, may be particularly vulnerable in schizophrenia and associated with negative symptoms and cognitive deficits [1,2,10]. Yet, most studies have relied on DTI measures, and NODDI-based characterization of callosal subregions—especially in first-episode schizophrenia (FES) patients—remains limited [6].

In the present study, we used NODDI to investigate microstructural alterations in the CC of FES patients compared to healthy controls (HCs). We first performed voxel-wise group comparisons of NDI and ODI within a CC mask, and then conducted region-of-interest (ROI) analyses in the GCC, BCC, and SCC. Motivated by our preliminary observations, we focused in particular on the SCC, where we noted: (i) significant group differences in NDI; (ii) no significant group difference in ODI; yet (iii) a robust association between SCC ODI and negative symptom severity, as measured by the Positive and Negative Syndrome Scale (PANSS). We hypothesized that, in the SCC, negative symptoms would be related to ODI conditional on a reduction in NDI, i.e., that orientation dispersion abnormalities would exert clinical relevance in the context of reduced neurite density.

To test this hypothesis, we examined: (1) group differences in NDI and ODI across CC sub-regions; (2) correlations between SCC NODDI metrics and PANSS scores; (3) the association between SCC ODI and symptoms after statistically controlling for NDI (residual ODI); and (4) the relationship between a composite index, the ODI/NDI ratio, and PANSS measures. By combining voxel-wise-based and ROI-based analyses, we aimed to delineate a more nuanced microstructural profile of the CC in early-stage schizophrenia and its link to negative symptoms.

## 2. Methods

### 2.1. Participants

This study was approved by the Institutional Review Board of the Second Affiliated Hospital, Zhejiang University School of Medicine. All participants provided written informed consent prior to enrollment. Thirty-two first-episode schizophrenia (FES) patients were recruited from the same hospital. Diagnosis was established by qualified clinical psychiatrists through structured clinical interviews based on DSM-5 (SCID) criteria. These patients were antipsychotic-naïve at recruitment. Symptoms were assessed by an experienced psychiatrist using the Positive and Negative Syndrome Scale (PANSS), a widely used clinical rating scale with demonstrated validity and reliability for measuring positive, negative, and general psychopathology [11]. Additionally, thirty age- and gender-matched healthy controls (HCs) were included in this study.

We recruited FES patients and demographically matched healthy controls (HCs). Inclusion criteria for FES patients included: (1) diagnosis of schizophrenia according to DSM-5 criteria, confirmed by structured clinical interview; (2) illness duration ≤ 18 months from the first psychotic episode. Exclusion criteria for all participants were: (1) a history of major neurological or medical disorders; (2) substance abuse or dependence within the past 6 months; (3) contraindications to MRI. HCs were additionally required to have no personal or family history (first-degree relatives) of psychotic disorders.

Demographic and clinical characteristics (age, sex, and PANSS scores) were summarized and compared between groups. A three-line table presents the group comparisons of demographic variables and PANSS scores (Table 1).

### 2.2. Imaging Acquisition

MRI data acquisition was performed on a Siemens 3T MAGNETOM Prisma system with a 20-channel head coil at Zhejiang University. Diffusion-weighted images (DWIs) were obtained using a simultaneous multi-slice (SMS) spin-echo echo-planar imaging prototype sequence [12,13]. The imaging parameters were as follows: repetition time (TR) = 3200 ms, echo time (TE) = 89 ms, field of view (FOV) = 210 mm × 210 mm, slice number = 92, voxel size = 1.5 mm × 1.5 mm × 1.5 mm, bandwidth = 1700 Hz/Px, and SMS factor = 4, with opposite phase-encoding directions along anteroposterior (AP) and posterior–anterior (PA) separately. The diffusion scheme contained three b-values (1000, 2000, 3000 s/mm^2^), with 30 vectors at each b-value, and six non-diffusion frames (b = 0). The total acquisition time (TA) was 10 min 56 s.

A T1-weighted anatomical image was also acquired by a magnetization-prepared gradient echo sequence with the following parameters: FOV = 240 mm × 240 mm, matrix = 256 × 256, slice number = 208, resolution = 0.9-mm isotropic, TR = 2300 ms, TE = 2.32 ms, inversion time = 900 ms, flip angle = 8°, TA = 5 min 21 s.

### 2.3. Image Processing

The DWI images were pre-processed using denoising, Gibbs-ring removal, and distortion and motion correction. The images along the AP and PA directions were combined for subsequent analysis. Additional pre-processing was carried out using the FSL Eddy tool [14] for motion and eddy current correction.

NODDI-derived parameters were estimated with the NODDI toolbox (www.nitrc.org/projects/noddi_toolbox (accessed on 7 May 2023)) [7]. The estimated parameters included the orientation dispersion index (ODI), the neurite density index (NDI), and free water fraction (FWF).

Subsequently, individual NODDI metric maps were nonlinearly registered to the FMRIB58 template using the TBSS pipeline implemented in FSL. Registration was visually inspected for accuracy in all subjects. The genu (GCC), body (BCC), and splenium (SCC) of the corpus callosum were defined using the JHU ICBM-DTI-81 white matter labels atlas in standard space. Mean values of ODI and NDI were then extracted from these three regions of interest for subsequent statistical analyses.

### 2.4. Z-Score Calculation

In order to quantify individual deviations in neurite density relative to the healthy reference distribution, we additionally computed voxel-wise *z*-scores of NDI (*z*_NDI) for each patient with schizophrenia. For every callosal ROI, the mean and standard deviation (SD) of NDI were first derived from the group of healthy controls. Individual patient *z-*scores were then calculated according to the following formula:

$${z\_NDI}_{subj}=\;\frac{{NDI}_{subj}-\mu_{HC}}{\sigma_{HC}}$$

where *z*_NDI denotes the standardized NDI score for each subject, ${NDI}_{subj}$ is the raw NDI value of the individual subject, and $\mu_{HC}$ and $\sigma_{HC}$ represent the mean and standard deviation of NDI in the healthy control group, respectively.

Thus, positive *z*-values indicate higher NDI relative to the control mean, whereas negative z-values indicate lower NDI relative to the control mean.

Corresponding *z*-scores were also calculated for ODI (*z*_ODI) using the same approach.

### 2.5. Statistical Analysis

Voxel-wise group comparisons of NODDI indices between patients and controls were performed within a corpus callosum mask using FSL “randomize” with 5000 permutations and threshold-free cluster enhancement (TFCE) to obtain family-wise error (FWE)-corrected *p* values [15,16]. Results were considered significant at *p*_FWE < 0.05.

For the region-of-interest (ROI) analyses, mean NDI and ODI values were extracted from the GCC, BCC, and SCC of the corpus callosum. Group differences in ROI-based NODDI metrics were assessed in GraphPad Prism 9.5.1 using two-tailed unpaired *t*-tests with Welch’s correction to account for potential inequality of variances between groups; *p* < 0.05 was considered statistically significant. Associations between ROI NODDI indices and PANSS scales were examined using two-tailed nonparametric Spearman rank correlations, given the non-normal distribution of some clinical variables. Additionally, linear regression analyses were performed to further evaluate the relationship between each ROI NODDI metric (NDI or ODI) and PANSS total/negative score, with age and sex included as covariates. The R^2^ and the fitted regression equation were reported for each regression model, and the regression curve was plotted with 95% confidence intervals and 95% prediction bands to visualize the linear trend. Age and sex were included as covariates in all analyses.

## 3. Results

### Demographic and Clinical Characteristics

FES patients and HC groups did not differ significantly in age, sex distribution. Detailed demographics and clinical measures are reported in Table 1.

- Voxel-wise NODDI Differences within the Corpus Callosum

**Figure 1:**
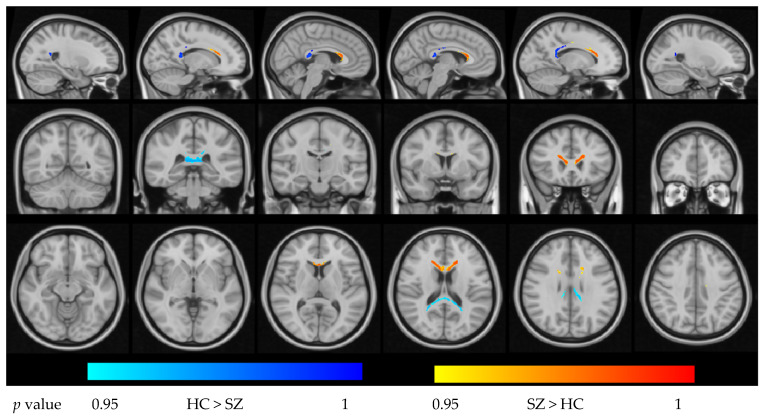
Group difference in NDI in the region of the corpus callosum (*p* < 0.05, FWE-corrected).

**Figure 2:**
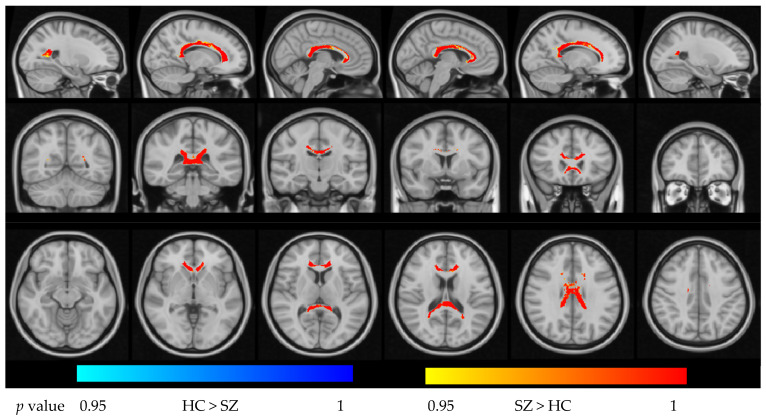
Group differences in ODI in the region of the corpus callosum (*p* < 0.05, FWE-corrected).

Within the CC mask, FES patients exhibited widespread increases in ODI relative to HCs in portions of the GCC, BCC, and SCC (*p* < 0.05, FWE-corrected). For the NDI, we observed a complex pattern: significantly reduced NDI in the posterior SCC but increased NDI in parts of the GCC/BCC in FES patients compared with HCs (*p* < 0.05, FWE-corrected). These effects are illustrated in Figure 1 and Figure 2.

b. ROI-based analysis of NDI and ODI in GCC, BCC, and SCC

**Figure 3:**
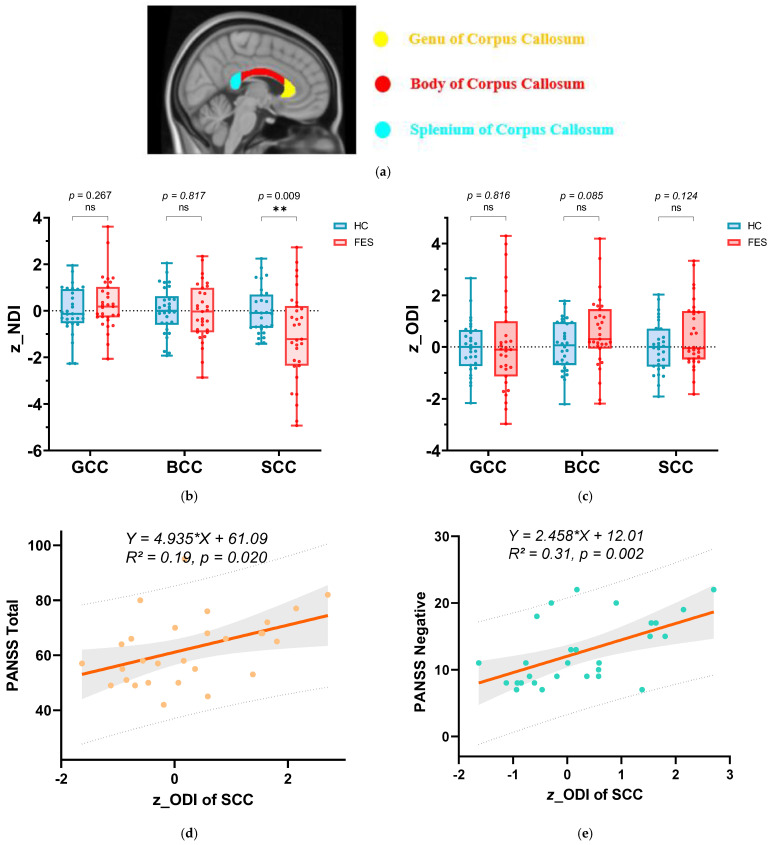
Intergroup differences in the three subregions of the corpus callosum between first-episode schizophrenia (FES) patients and healthy controls and their correlation with the PANSS scale. (**a**) The three subregions of the corpus callosum: the genu of corpus callosum (GCC), the body of corpus callosum (BCC), and the splenium of corpus callosum (SCC). (**b**) Intergroup differences in z-scores of NDI for the three subregions. (**c**) Intergroup differences in *z*-scores of ODI for the three subregions. Group comparisons of (**b**,**c**) were performed using two-tailed unpaired *t*-tests with Welch’s correction. *p*-values are shown above the comparisons. ** represents *p *< 0.01. “ns” stands for not significant. Blue indicates healthy controls. Red indicates FES patients. (**d**) Linear regression curve between *z*_ODI in the SCC and PANSS total scores. (**e**) Linear regression curve between *z*_ODI in the SCC and PANSS negative scores. In the plots of (**d**,**e**), the solid line represents the regression line, with the shaded area indicating the 95% confidence interval, and the dashed lines indicating the 95% prediction interval. The fitted regression equation and R^2^ value are shown on the graph.

In the ROI-based analysis, we first compared the mean *z*_NDI and *z*_ODI between the FES patients and HCs within the GCC, BCC, and SCC (Figure 3). Consistent with the voxel-wise findings, z_NDI in the SCC was significantly reduced in FES patients compared with HCs (*p* = 0.009), indicating a marked decrease in neurite density in the posterior corpus callosum, whereas no significant group differences in NDI were observed in the GCC or BCC. In contrast, z_ODI did not differ significantly between groups in the SCC (*p* = 0.124), suggesting that, at the regional level, overall fiber orientation dispersion in this segment of the callosum was relatively preserved despite reduced neurite density.

Although group-level differences in SCC ODI were nonsignificant, individual variability in this metric was strongly related to symptom severity in FES patients. Higher z_ODI in the SCC was positively correlated with higher PANSS negative scores (*r* = 0.554, *p* = 0.002) and PANSS total scores (*r* = 0.457, *p* = 0.014) using Spearman correlation. Figure 3d,e depict the linear regression curves between *z*_ODI in the SCC and PANSS total scores (3d, R^2^ = 0.19) and PANSS negative scores (3e, R^2^ = 0.31), with 95% confidence intervals and prediction bands.

Importantly, these associations remained significant after regressing NDI out of ODI (residual *z*_ODI). Higher *z*_ODI in the SCC was positively correlated with higher PANSS negative scores (*r* = 0.503, *p* = 0.006) and PANSS total scores (*r* = 0.474, *p* = 0.011), as assessed by Spearman correlation.

**Figure 4:**
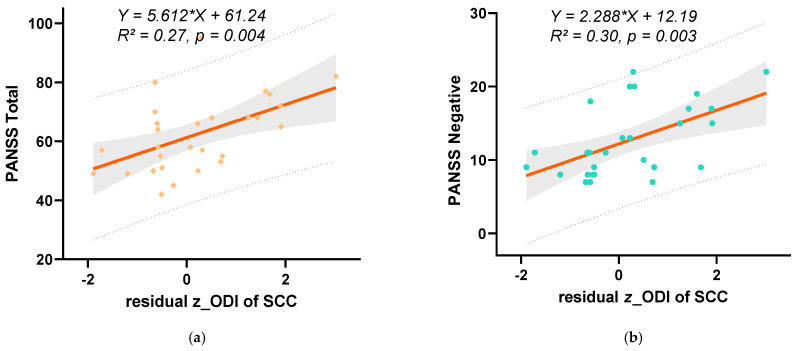
Linear regression curves between indicators of the SCC and PANSS scale scores. (**a**) The linear regression curve between the z-score of ODI after regressing out NDI and PANSS total score. (**b**) Linear regression curve between z score of ODI after regressing out NDI and PANSS negative scores. In the plots of (**a**,**b**), the solid orange line represents the regression line, with the shaded area indicating the 95% confidence interval, and the dashed lines indicating the 95% prediction interval. The fitted regression equation and R^2^ value are shown on the graph.

Figure 4a,b show the linear regression curves between residual *z*_ODI in the SCC and PANSS total scores (4a, R^2^ = 0.27) and the PANSS negative scale (4b, R^2^ = 0.30), again with 95% confidence intervals and prediction bands.

**Figure 5:**
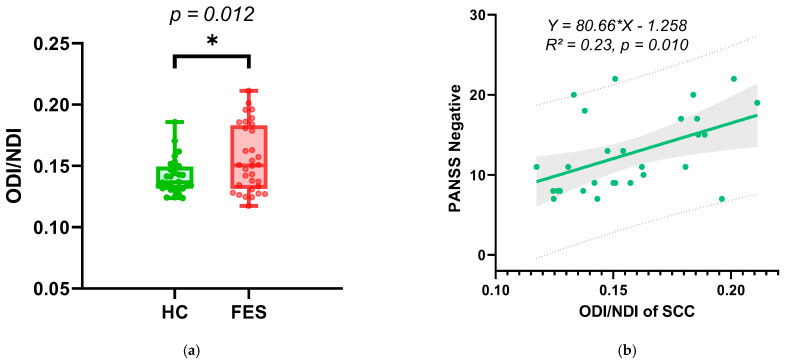
(**a**) Intergroup comparison of ODI/NDI in the SCC: FES patients versus healthy controls assessed using two-tailed unpaired *t*-tests with Welch’s correction. The p-value is indicated above the comparison (*p* = 0.012). * represents *p* < 0.05. (**b**) Thee linear regression curve between ODI/NDI and PANSS negative scores. In the plot, the solid line represents the regression line, with the shaded area indicating the 95% confidence interval and the dashed lines indicating the 95% prediction interval. The fitted regression equation and R^2^ value are shown on the graph.

Figure 5a illustrates the between-group comparison of ODI/NDI in the SCC (FES patients versus healthy controls) using two-tailed unpaired *t*-tests with Welch’s correction (*p* = 0.012). Figure 5b presents the linear regression curves between ODI/NDI in the SCC and PANSS negative scores (R^2^ = 0.30). The composite indicator ODI/NDI measure in the SCC was positively correlated with PANSS negative scores (*r* = 0.457, *p* = 0.014) using Spearman correlation.

These results indicate that, under conditions of reduced neurite density in the SCC, greater fiber orientation dispersion—captured by ODI-based composite indices—reflects clinically relevant microstructural disorganization linked specifically to negative symptoms in first-episode schizophrenia.

## 4. Discussion

In this NODDI study of FES patients, regionally specific microstructural alterations of the corpus callosum (CC) were identified and shown to have distinct relationships with clinical symptoms. At the voxel-wise level, FES patients showed widespread increases in ODI across the genu (GCC), body (BCC), and splenium (SCC), together with a heterogeneous pattern of NDI changes, characterized by reduced NDI in the posterior SCC and increased NDI in portions of the GCC/BCC, consistent with prior NODDI work in antipsychotic-naïve FEP showing decreased ND in commissural/association fibers, with increased ND in projection fibers and largely increased ODI across fiber types [17,18]. ROI analyses confirmed significantly reduced SCC NDI in FES patients, whereas mean SCC ODI did not differ between groups. Notably, SCC ODI and derived indices (residual ODI after regressing out NDI and the ODI/NDI ratio) were robustly associated with PANSS negative and total scores, while SCC NDI was not. These findings suggest that, in early-stage schizophrenia, negative symptoms are preferentially linked to neurite orientation dispersion under conditions of reduced neurite density in posterior callosal fibers.

### 4.1. Comparison with Previous Diffusion and NODDI Findings

Abnormalities of the CC are consistently reported in schizophrenia, particularly reduced FA and altered diffusivity in DTI studies of both chronic and early-stage patients [19]. Meta-analytic and region-of-interest studies have highlighted prominent involvement of the SCC and GCC, which contain fibers interconnecting the occipital, parietal, and frontal regions, which support higher-order integration [19,20,21]. Our observation of reduced NDI in the SCC is in line with previous DTI work and, by leveraging NODDI, provides a more biologically specific interpretation of previous FA reductions in terms of decreased axonal packing or myelination rather than nonspecific “white matter damage” [7].

NODDI has been increasingly used to investigate microstructural abnormalities in psychiatric disorders and has proven sensitive to subtle gray and white matter microstructural abnormalities [22]. A systematic review across major psychiatric disorders (schizophrenia, bipolar disorder, depression, autism, and ADHD) concluded that NODDI consistently detects alterations in NDI and ODI, with variable spatial patterns and frequent associations with symptom domains [23]. In first-episode psychosis, Rae et al. reported reduced NDI in multiple commissural and association tracts, including the CC, and showed that FA abnormalities largely co-localized with decreased NDI rather than aberrant fiber orientation [18]. Kraguljac et al. further demonstrated in antipsychotic-naïve FEP that ND is decreased in commissural and association fibers but relatively increased in projection fibers, whereas ODI is largely increased across fiber types [17]. Our mixed pattern of NDI alterations—reduced in SCC but increased in anterior callosal segments—accords with this notion of tract- and region-dependent microstructural changes and suggests that callosal pathology in schizophrenia is not spatially homogeneous [17,18,23].

Beyond psychosis, NODDI has demonstrated that reduced NDI and/or altered ODI are sensitive markers of white matter pathology in a range of neurological and psychiatric conditions, often providing additional information beyond DTI. For example, decreased NDI in commissural and association tracts is prominent in autism and relates to behavioral features, while reduced NDI in the forceps major and associated visual association areas is linked to deficits in perceptual integration and facial emotion recognition. In minimal hepatic encephalopathy and mild traumatic brain injury, NDI reductions in the CC, particularly the SCC, are closely related to cognitive deficits, and NODDI-derived indices outperform DTI in detecting these associations [24,25]. Consistent with these reports, our finding of SCC NDI reduction aligns with the view that apparent axonal density is a core microstructural factor in callosal abnormalities across diverse conditions.

Biologically, higher NDI in anterior callosal regions in FES may reflect altered neurodevelopmental trajectories of frontal interhemispheric fibers. Anterior segments of the CC, which connect prefrontal and anterior cingulate cortices, undergo protracted maturation and myelination into early adulthood. In the context of reduced NDI in the SCC, relatively increased NDI in GCC/BCC could index either incomplete or aberrant pruning, or region-specific myelination changes of frontal callosal axons, consistent with NODDI reports of increased neurite density in projection pathways in early psychosis. Rather than indicating a simple enhancement of connectivity, such anterior NDI increases may represent a compensatory or maladaptive reorganization of callosal architecture that co-occurs with posterior fiber loss in early schizophrenia.

### 4.2. Functional and Clinical Interpretation of Splenial Abnormalities

The SCC primarily contains fibers connecting bilateral occipital and posterior parietal/temporal regions involved in visual information transfer, visuospatial integration, and social-cognitive processing [26,27,28]. NODDI work in autism has demonstrated that microstructural disturbances in the forceps major and ventral occipitotemporal regions are associated with impaired interhemispheric integration of facial emotional information and communication difficulties [29]. Similarly, decreased NDI in callosal and long-range association pathways has been related to core social and cognitive symptoms in autism and other neurodevelopmental disorders [29]. Our observation that SCC NDI is reduced already in FES patients is therefore consistent with an early disruption of posterior interhemispheric connectivity that may contribute to cognitive and socio-affective disturbances in schizophrenia.

The key novel contribution of this study lies in the dissociation between mean SCC ODI (no group difference) and its strong association with negative symptom severity when considered together with NDI. This pattern suggests that between-group comparisons of ODI alone may underestimate clinically relevant variability. Instead, ODI appears to capture individual differences in the degree of residual fiber orientation disorganization among patients who already share a common background of reduced neurite density. The fact that residual ODI (controlling for NDI) and the ODI/NDI ratio show stronger correlations with negative and total PANSS scores than either NDI or ODI alone indicates that the balance between neurite dispersion and density is particularly important for clinical expression. A higher ODI/NDI ratio in the SCC may represent sparse but highly disorganized callosal fibers, leading to inefficient or noisy interhemispheric signaling, which in turn could manifest as blunted affect, avolition, and social withdrawal.

Such a density–dispersion imbalance has analogues in other disorders. In youth with congenital heart disease, lower NDI (with relatively preserved ODI) in the CC and long association tracts is thought to reflect enduring consequences of early white matter dysmaturation that contribute to neuropsychological difficulties. In mild TBI, NDI in the SCC is tightly linked to processing speed and global cognition [24]. Our results extend these observations to early schizophrenia and specifically implicate THE posterior callosal microstructure—quantified by composite NODDI indices—as a substrate of negative symptoms, a clinical dimension strongly associated with functional outcomes and often refractory to current treatments.

### 4.3. Pathophysiological Implications

Converging imaging and postmortem evidence points to microstructural involvement—axonal and dendritic changes, oligodendroglial dysfunction, and synaptic loss—as a core pathophysiological feature of schizophrenia [23,30,31]. Advanced diffusion models such as NODDI are thought to provide a closer in vivo proxy of neurite density and orientation, complementing structural MRI and synaptic PET markers of reduced synaptic density. In this context, SCC NDI reductions in FES patients are consistent with a neurodevelopmental model, in which aberrant myelination and axonal pruning during adolescence lead to long-lasting dysconnectivity, particularly in large commissural tracts [17,18,31].

The link between negative symptoms and orientation dispersion-based indices in the SCC suggests that, beyond loss of fibers, maladaptive reorganization and disordered geometry of the remaining neurites may be critical for symptom expression. Longitudinal NODDI work in schizophrenia has shown that whole-brain ODI abnormalities can be present prior to or independent of major changes in free water, and that lower fiber uniformity may predict poor treatment response [19]. Together with our findings, this raises the possibility that ODI-related measures reflect a dimension of “microstructural instability” or inefficient network organization that is relatively insensitive to short-term pharmacological modulation but closely tied to persistent negative symptoms and social-cognitive deficits [9,19,20,23].

NODDI studies in other diseases also support a dissociation between density and dispersion: in multiple sclerosis, NDI is consistently reduced in lesions and normal-appearing white matter, whereas ODI changes are more variable and may reflect complex remodeling of surviving fibers [31,32,33]. In animal and translational models relevant to psychiatric illness, decreased NDI in major white matter pathways is interpreted as axonal loss, while ODI alterations in cortical regions suggest concomitant dendritic change [34,35]. Collectively, these data support a model in which NDI indexes the structural substrate (number/packing of neurites), whereas ODI and composite measures (e.g., ODI/NDI) capture organization and complexity of remaining fibers, both of which may have distinct clinical correlates. In parallel, the relative increase of NDI in anterior callosal segments may reflect region-dependent neurodevelopmental alterations of frontal interhemispheric fibers, potentially representing compensatory or maladaptive changes that accompany posterior splenial neurite loss.

The corpus callosum is composed predominantly of long-range glutamatergic pyramidal axons that interconnect homologous cortical areas. In this context, reduced NDI and an increased dispersion–density imbalance in the SCC may reflect a disturbed interhemispheric glutamatergic drive between posterior temporal–parietal–occipital regions. Such disruption of transcallosal excitatory input could secondarily impact cortico–striato–thalamo–cortical circuits that regulate midbrain–striatal dopaminergic tone, thereby providing a mechanistic link between white matter microstructural abnormalities and the dopamine hypothesis of schizophrenia. While our data are not designed to test this model directly, they are consistent with a scenario in which aberrant callosal glutamatergic transmission contributes to dysregulated dopaminergic signaling and the emergence of negative symptoms against a background of impaired large-scale network integration.

It should be emphasized that the SCC, like other callosal segments, contains not only myelinated axons but also oligodendrocytes and other glial populations. Post-mortem and imaging work indicates that oligodendroglial dysfunction is a key component of schizophrenia-related microstructural pathology, which may contribute to both axonal abnormalities and altered myelination within the corpus callosum. Moreover, recent experimental findings implicating trace-amine-associated receptor 1 (TAAR1) signaling in glial cells suggest that TAAR1-related pathways may modulate white matter integrity and represent a potential therapeutic avenue in patients with schizophrenia [36]. Although NDI and ODI provide neurite-centered estimates and cannot directly separate axonal from glial contributions, glial abnormalities (including disturbed TAAR1-mediated signaling) could plausibly lead to changes in axonal packing, myelination, and extracellular milieu that manifest as the SCC density–dispersion imbalance observed here. Future multimodal work combining NODDI with glia-sensitive molecular imaging will be necessary to delineate these cellular contributions more precisely.

### 4.4. Methodological Considerations

Several methodological aspects strengthen the current findings. First, focusing on first-episode patients reduces confounds of chronicity and long-term antipsychotic exposure and aligns with other early-psychosis NODDI studies that report widespread NDI reductions and ODI increases [17,18,37]. Second, we used a multi-shell diffusion acquisition and the original NODDI toolbox, which is recommended for reliable estimation of NDI and ODI, and is consistent with technical recommendations from recent systematic reviews [6,17,19]. Third, restricting the analysis to a CC mask and subdividing it into the GCC, BCC, and SCC allowed us to detect regionally specific patterns that may be diluted in whole-brain approaches. Finally, our use of z-scored NDI/ODI, residual ODI, and the ODI/NDI ratio parallels approaches in other clinical NODDI studies, where composite or normalized indices proved more tightly linked to behavioral or cognitive outcomes than single parameters.

### 4.5. Limitations

One limitation of this study is that the PANSS was administered only to patients with first-episode schizophrenia. The PANSS is a disease-specific instrument validated for schizophrenia populations and is not appropriate for use in healthy controls, as it assesses symptoms that are typically absent in individuals without psychopathology. Consequently, it is not possible to directly compare PANSS scores between groups.

The apparent dissociation between the absence of a significant group difference in mean SCC ODI and its robust association with negative symptoms should be interpreted with caution in light of the modest sample size. With limited statistical power, group-level comparisons may fail to detect small to moderate differences, whereas within group correlations can still emerge if inter individual variability in ODI is substantial. An alternative, non-mutually exclusive explanation is that SCC orientation dispersion is genuinely heterogeneous among FES patients, such that only a subset with pronounced dispersion abnormalities exhibits severe negative symptoms. Larger, ideally multi-site studies are required to determine whether the density–dispersion imbalance observed here is stable and generalizable.

The sample size was modest, which may have limited the power to detect symptom correlations in the GCC and BCC and could contribute to Type II error. The effects of antipsychotic medication cannot be completely excluded, although NODDI studies in antipsychotic-naïve and unmedicated patients also report microstructural abnormalities and limited short-term normalization with treatment. NODDI parameters are model-based estimates and remain influenced by factors such as free water content, partial volume, and crossing fibers, despite explicit modeling of an isotropic compartment [6]. Finally, the cross-sectional design precludes causal inference regarding whether SCC microstructural disturbances precede or follow the onset of negative symptoms [38].

It should be emphasized that NODDI-derived indices are model-based MRI measures and do not constitute direct histological evidence; inferences regarding axonal and dendritic changes remain inferential and require confirmation with complementary modalities.

### 4.6. Future Directions

Future studies should adopt longitudinal designs to track trajectories of NDI, ODI, and composite indices from the high-risk/prodromal stage through the first-episode and chronic phases, and to test whether SCC ODI/NDI measures predict long-term negative symptom burden or treatment response, in line with initial longitudinal evidence that NODDI metrics can predict antipsychotic response and evolve with the illness course. Integrating NODDI with detailed cognitive and social functioning assessments, as well as functional MRI, would clarify how splenial density–dispersion imbalance impacts functional connectivity and network efficiency in posterior–frontal circuits. Expanding analyses to other white matter pathways and combining NODDI with complementary microstructural models (e.g., free-water imaging, diffusional kurtosis) may further refine the characterization of white matter pathology in schizophrenia. Finally, multi-modal work linking NODDI with molecular or synaptic imaging (e.g., SV2A PET) could help bridge the gap between microstructural diffusion metrics and underlying cellular and synaptic changes.

## 5. Conclusions

Using neurite orientation dispersion and density imaging in first-episode schizophrenia, this study demonstrates that the corpus callosum exhibits regionally specific microstructural alterations, with reduced neurite density in the splenium and increased orientation dispersion across callosal sub-regions. A key novel observation is that, within the SCC, negative symptom severity is not simply related to neurite loss but rather to the degree of neurite orientation disorganization against a background of reduced neurite density. Composite indices such as residual ODI (controlling for NDI) and the ODI/NDI ratio showed stronger and more specific associations with negative and total PANSS scores than either NDI or ODI alone.

These results complement prior NODDI work in first-episode psychosis and other neuropsychiatric conditions and highlight the added value of biologically informed diffusion models for capturing clinically relevant white matter abnormalities that may be missed by conventional DTI [6,39]. Posterior callosal microstructure, quantified by NODDI-derived density–dispersion metrics, may represent a promising imaging biomarker for negative symptoms in early schizophrenia and a potential target for interventions aimed at preserving or restoring efficient interhemispheric connectivity.

## Figures and Tables

**Table 1:** Demographic and psychopathological data.

	HC	FES
Number of Subjects	30	32
Age	21.2 ± 4.6	20.5 ± 5.2
Gender (f, m)	14, 16	17, 15
Handedness (left/right)	0/30	0/32
PANSS Positive	/	15.61 ± 5.31
PANSS Negative	/	12.64 ± 4.92
PANSS Total	/	62.36 ± 12.54

HCs, healthy controls; FES, first-episode schizophrenia patients; f, female; m, male; PANSS, positive and negative syndrome scale.

## Data Availability

Data will be made available on reasonable request and with approval from the affiliated institutions.

## Abbreviations

The following abbreviations are used in this manuscript:

BCC	Body of corpus callosum
CC	Corpus callosum
DWI	Diffusion-weighted imaging
FES	First-episode schizophrenia
FWE	Family-wise error
GCC	Genu of corpus callosum
HC	Healthy control
MRI	Magnetic Resonance Imaging
NDI	Neurite density index
NODDI	Neurite orientation dispersion and density imaging
ODI	Orientation dispersion index
PANSS	Positive and Negative Syndrome Scale
ROI	Region of interest
SCC	Splenium of corpus callosum
SD	Standard deviation
SZ	Schizophrenia
